# Antinociceptive effect of plant-based natural products in chemotherapy-induced peripheral neuropathies: A systematic review

**DOI:** 10.3389/fphar.2022.1001276

**Published:** 2022-09-19

**Authors:** Wagner Barbosa Da Rocha Santos, Juliana Oliveira Guimarães, Lícia Tairiny Santos Pina, Mairim Russo Serafini, Adriana Gibara Guimarães

**Affiliations:** ^1^ Graduate Program in Pharmaceutical Sciences, Department of Pharmacy, Federal University of Sergipe, São Cristóvão, Sergipe, Brazil; ^2^ Graduate Program in Health Sciences, Federal University of Sergipe, São Cristóvão, Sergipe, Brazil

**Keywords:** natural product, plants, pain, antinociceptive, chemotherapy, peripheral neuropathies

## Abstract

Chemotherapy-induced peripheral neuropathy (CIPN) is one of the most prevalent and difficult-to-treat symptoms in cancer patients. For this reason, the explore for unused helpful choices able of filling these impediments is essential. Natural products from plants stand out as a valuable source of therapeutic agents, being options for the treatment of this growing public health problem. Therefore, the objective of this study was to report the effects of natural products from plants and the mechanisms of action involved in the reduction of neuropathy caused by chemotherapy. The search was performed in PubMed, Scopus and Web of Science in March/2021. Two reviewers independently selected the articles and extracted data on characteristics, methods, study results and methodological quality (SYRCLE). Twenty-two studies were selected, describing the potential effect of 22 different phytochemicals in the treatment of CIPN, with emphasis on terpenes, flavonoids and alkaloids. The effect of these compounds was demonstrated in different experimental protocols, with several action targets being proposed, such as modulation of inflammatory mediators and reduction of oxidative stress. The studies demonstrated a predominance of the risk of uncertain bias for randomization, baseline characteristics and concealment of the experimental groups. Our findings suggest a potential antinociceptive effect of natural products from plants on CIPN, probably acting in several places of action, being strategic for the development of new therapeutic options for this multifactorial condition.

## Graphical Abstract

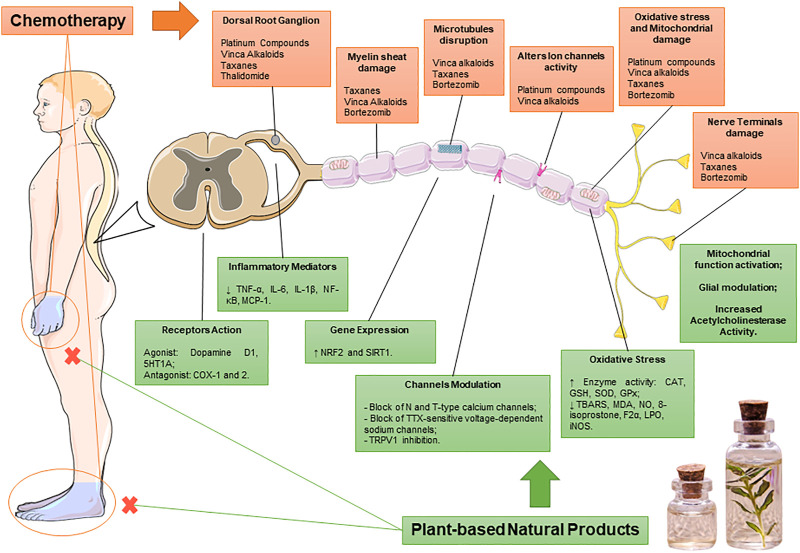

## 1 Introduction

Chemotherapy-induced peripheral neuropathy (CIPN) accounts for the most common neurological complication of cancer treatment with reporting rates ranging from 19% to more than 85%, presenting an agent-based prevalence (Zajączkowska et al., 2019). Chemotherapeutic agents such as paclitaxel, oxaliplatin, docetaxel, bortezomib and thalidomide are often involved in a sensory peripheral neuropathy that manifests symptoms as cold and mechanical allodynia, paresthesia, numbness and tingling in the feet and hands (“stocking and glove” distribution) (Hou et al., 2018; Wu et al., 2019).

In this sense, the development of neuropathies is the most recurrent cause for alteration of chemotherapy regimens, leading to a decrease in dose and frequency or choice of a different therapeutic agent, leading to impairments to the treatment and a lower quality of life (Lee et al., 2021). Unfortunately, there are no sufficient options available for the CIPN treatment (Colvin, 2019).

In fact, although being considered as an neuropathic pain, current pharmacological treatments for this condition (tricyclic antidepressants, anticonvulsants, etc.) are slightly effective in CIPN or have undesirable reactions (Hou et al., 2018), such as dizziness, nausea, somnolence and vomiting (Lee et al., 2021). Therefore, identifying alternative classes of analgesics that can effectively manage pain in cancer patients is of great importance (Blake et al., 2017).

In this perspective, natural products still stand out as the most important and valuable source of therapeutic agents, as they present a vast number of bioactive chemical compounds (Lautié et al., 2020; Newman and Cragg, 2020; Singh Aidhen and Thoti, 2021). In fact, about one third of the best-selling drugs in the word are natural products or their derivatives. Besides, it is estimated that more than 35% of medicines used worldwide were originated directly or indirectly from natural products, making them an important resource for global pharmaceutical companies working on the development of new medicines (Calixto, 2019). However, only less than 15% of natural sources have been explored so far; among them, those derived from plants have a wide chemical and structural diversity, representing the most prevailing source for the detection of novel medicinal substances (Thomas et al., 2021).

Therefore, considering the imminent need for new therapeutic options for chemotherapy-induced peripheral neuropathy and the analgesic and anti-inflammatory potential of plant-based natural products, this review aims to report the effect of compounds of this origin, and how they would be acting to reduce CIPN.

## 2 Methods

### 2.1 Focused question

The key question to be answered by the development of this review was: Are natural products from plants capable of controlling peripheral neuropathy caused by chemotherapy?

### 2.2 Protocol and registration

The Preferred Reporting Items for Systematic Reviews and Meta-Analyses (PRISMA) tool was used as a guide for the construction of the review (Page et al., 2021). It was registered in the international database of systematic reviews of PROSPERO obtaining the registration number CRD42021242592 and the protocol is available at https://www.crd.york.ac.uk/PROSPERO/display_record.php?RecordID=242592.

### 2.3 PICOS strategy

This review was carried out according to the PICOS strategy (Santos et al., 2007), detailing the following information: P (problem): peripheral neuropathy caused by chemotherapeutic drugs; I (intervention): treatment with plant-based natural products; C (control): without treatment or standard drugs; O (outcomes): nociceptive responses in animals with chemotherapy-induced peripheral neuropathy; S (type of study): pre-clinical studies.

### 2.4 Search strategy and literature search

The research was carried out in March 2021, using PubMed, Web of Science and Scopus databases. The research strategy was carried out using different combinations of the following MESH descriptors, in addition to their synonyms: “chemotherapy-induced peripheral neuropathy, CIPN, (peripheral neuropathy and cancer),” and “plant-based natural products, terpene, flavonoid, coumarin, xanthone, chromone, lignan, neolignan, tannin, saponin, alkaloid, xanthine.” The search strategy is detailed in the Supplementary Table S1.

### 2.5 Selection of studies and eligibility criteria

To select the studies, two reviewers (W.B.R.S. and L.T.S.P.) independently evaluated the results of the research using an application to aid in the preparation of systematic reviews, Rayyan (Ouzzani et al., 2016). After reading the titles and abstracts, potentially relevant studies were selected to read the full text according to the eligibility criteria and the agreement between these reviewers was measured using the Kappa statistical test (Landis and Koch, 1977). Disagreements were resolved by consensus or by consulting a third reviewer (A.G.G.).

The following eligibility criteria were used: 1) articles with descriptors and/or synonyms in the title, abstract or full text; 2) studies with isolated compounds from medicinal plants evaluating the antinociceptive effect against peripheral neuropathies induced by chemotherapy drugs; 3) preclinical studies. In addition, studies were selected without language and year restriction. Studies using the association of natural products with other substances and modification of the chemical structure of natural products, review articles, duplicates, abstracts, clinical trials and *in vitro* studies were excluded. The reference lists of the selected articles were searched manually to identify other studies that could meet the inclusion criteria and were not found in the databases searched.

### 2.6 Data extraction and risk of bias assessment

The following information was extracted from the selected studies: name of the plant-based natural product, means of obtaining, model of induction of peripheral neuropathy, evaluated methods, dosage and route of administration, positive control used or not, the type of animal used and the results obtained by the study. In addition, the authors of the selected studies were contacted when the articles or data were unavailable.

The methodological quality of the studies was assessed using the SYRCLE risk of bias protocol for animal studies. Ten domains were evaluated, which include: selection bias (sequence generation, allocation hiding and random accommodation), performance bias (random accommodation and concealment), detection bias (evaluation and hiding random results), attrition bias (data incomplete results), report bias (selective results report) and other sources of bias, such as inadequate influence from funders. The risk of bias was classified as low, uncertain or high, according to the established criteria (Hooijmans et al., 2014).

## 3 Results and discussion

**FIGURE 1:**
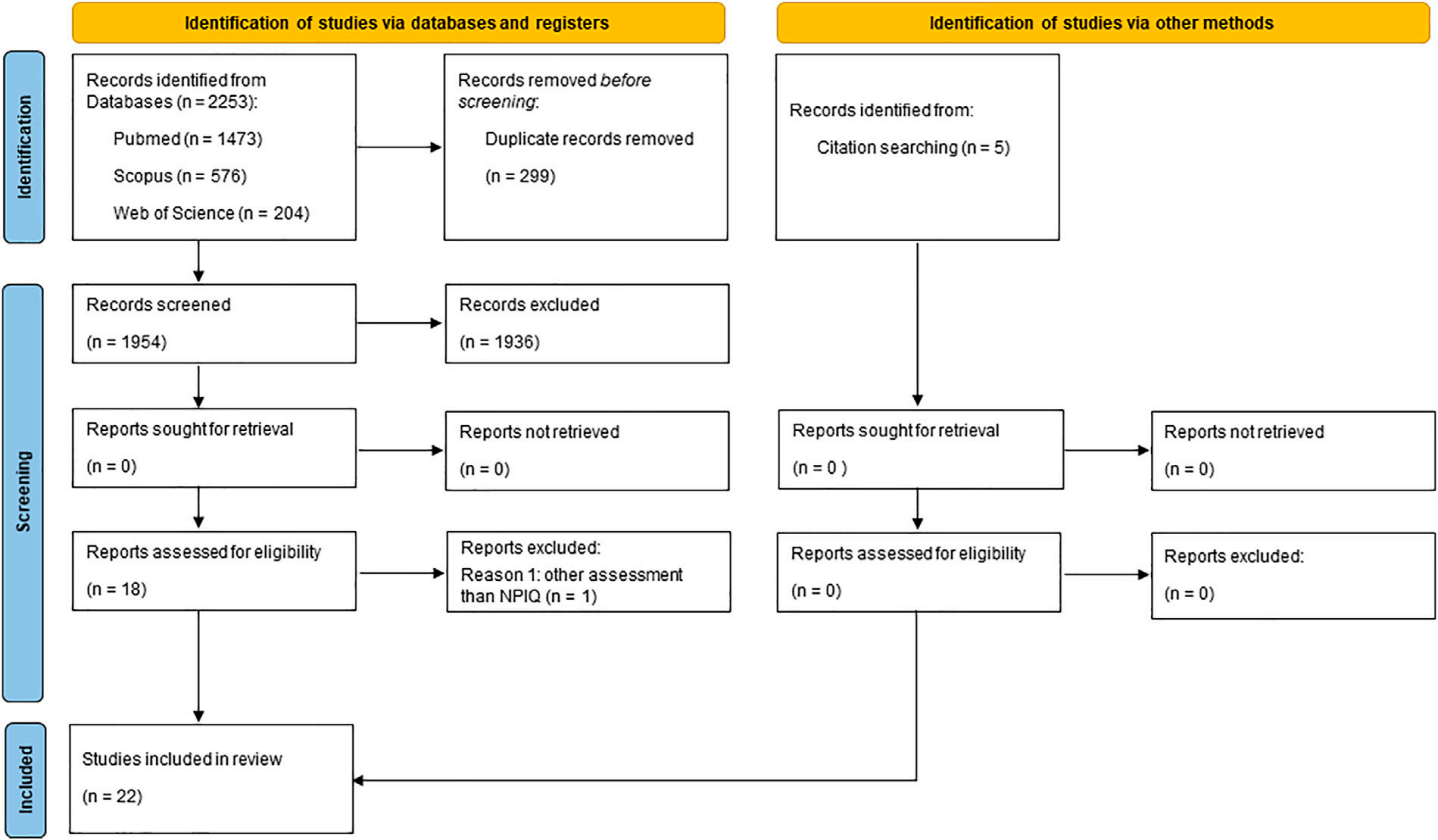
Flowchart of included studies.

The present study carried out a survey of publications on compounds isolated from plant-based natural products and peripheral neuropathy induced by chemotherapy, with 1,473 articles found in the PubMed database, 576 in Scopus and 204 articles in the Web of Science, totalizing 2,253 articles. After excluding duplicate articles (299), 1,954 studies were submitted to the reading of titles and abstracts, based on the established inclusion and exclusion criteria; 1,936 articles were excluded and 18 were selected for the full reading of the text. Only one study was excluded after reading the full text because it did not represent a model of chemotherapy-induced peripheral neuropathy. In addition, after manually searching the list of references for the included studies, five more studies were added, totalizing 22 articles (Figure 1). Kappa agreement between the evaluators was 0.748, considered as substantial (0.6–0.79) (Landis and Koch, 1977).

### 3.1 Overview of included studies

**FIGURE 2:**
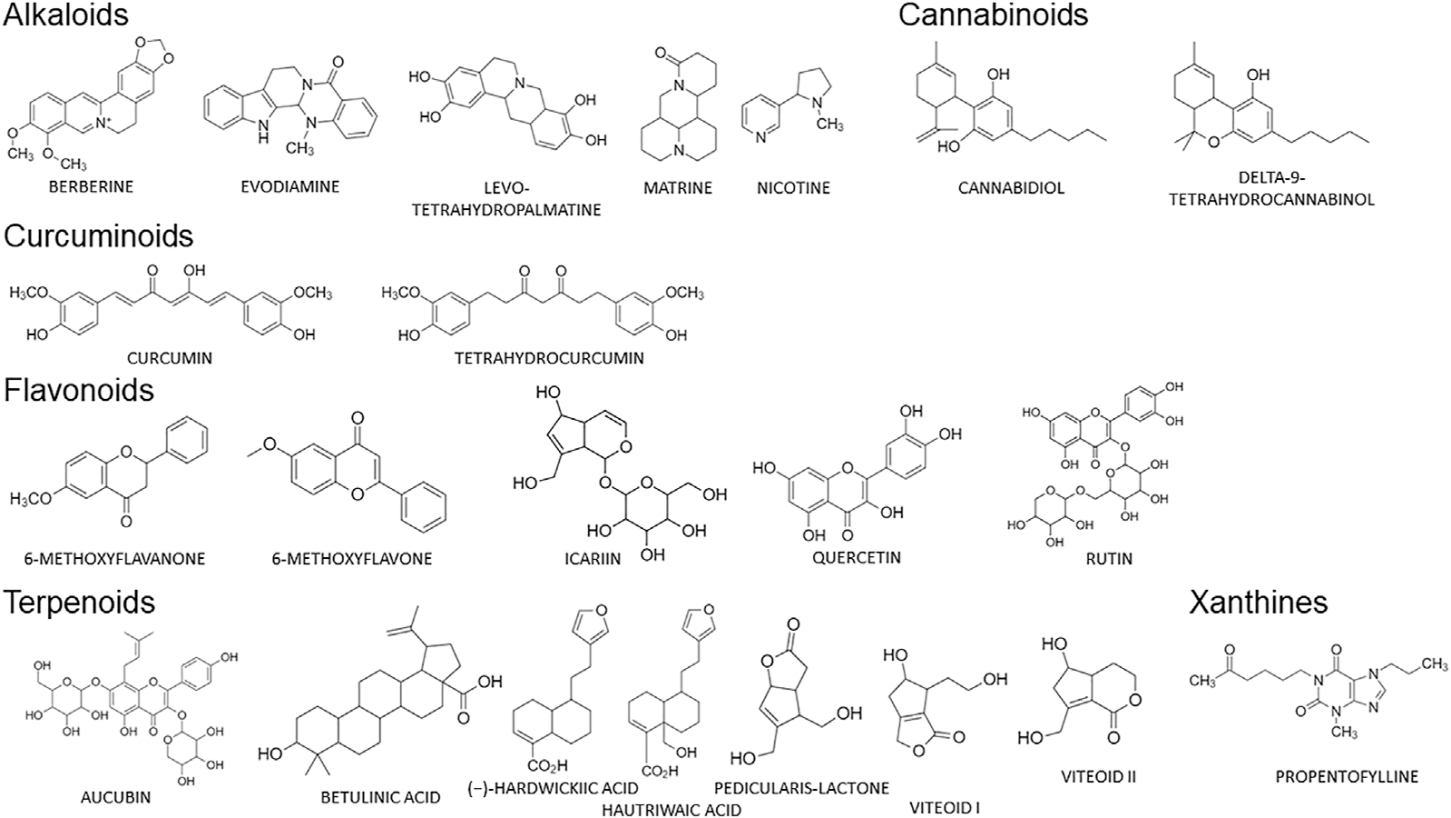
Molecular structures classified by secondary metabolites of all substances included in this systematic review.

A summary of each study is presented in Table 1. Different compounds from plants, animals, methods of induction and pain assessment were used by the selected studies. Studies with 22 different compounds were found (Figure 2) and the same compound may be presented in different articles. These compounds can be divided into the following classes of secondary metabolites: Tepernes (*n* = 7, 31.81%) Alkaloids (*n* = 5, 22.73%), Flavonoids (*n* = 5, 22.73%), Cannabinoids (*n* = 2, 9.09%), Curcuminoids (*n* = 2, 9.09%), and Xanthines (*n* = 1, 4.55%). These findings corroborate with Gouveia et al. (2019), who demonstrated that compounds belonging to the class of terpenes, alkaloids, flavonoids and cannabinoids have been the target of recent studies on their effects on cancer pain. In fact, the analgesic properties of compounds belonging to these classes of natural products have been extensively demonstrated in several studies, due to their ability to modulate neurotransmission systems, inflammatory response and redox imbalance (Guimarães et al., 2014; Gouveia et al., 2018; Ferraz et al., 2020; Zhu et al., 2020; Anand et al., 2021).

**TABLE 1:** Description of the chemical and pharmacological aspects of the preclinical studies included in the systematic review.

Substance	Obtaining	Model	Evaluation methods	Dose (*via*)	Animal	Positive control	Results	Other outcomes	References
Alkaloids									
Berberine	Sigma Aldrich Ltd. (United States)	PTX (1 mg/kg, i.p. on alternate days for 4 days—4 mg/kg cumulative dose)	Thermal hyperalgesia and Cold allodynia (tail flick latency)	10 or 20 mg/kg, i.p.	Male Wistar rats	Amytriptiline (10 mg/kg, i.p.)	**Thermal hyperalgesia:** inhibited decrease of latency in tail flick test **Cold allodynia:** no significant decrease in tail cold allodynia latencies	Increased expression of the NRf2 gene and antioxidant activity (decreased TBARS and increased SOD, GPx and GSH)	Singh et al. (2019)
Evodiamine	Kisshida Chemical Co., Ltd. (Osaka, Japan)	PTX (1 mg/kg, i.p., on alternate days for 4 days—4 mg/kg cumulative dose)	Mechanical allodynia (von Frey filaments) and Thermal hyperalgesia (hot plate test)	5 mg/kg, i.p.	Male Sprague–Dawley rats	Not reported	Decreased the thermal sensitivity and inhibited the reduction of the hind paw withdrawal	Activation of mitochondrial function, decreased TNF-α, IL-1β, IL-6, MCP-1 and antioxidant activity (decreased MDA, NO and 8-isoprostane F2α)	Wu and Chen (2019)
Levo-tetrahydropalmatine	Shanghai Lei Yun Shang Pharmaceutical Co.	OXL (3 mg/kg, i.p., 5 consecutive days for 2 weeks with a total of 10 injections—30 mg/kg cumulative dose)	Mechanical hyperalgesia (von Frey filaments)	1, 2 e 4 mg/kg, i.p.	Male C57BL/6 mice	Not reported	Increased the paw withdrawal threshold in mice	D1 dopamine receptor agonist	Guo et al. (2014)
Matrine	(Ningxia Zi Jing Hua Pharmacy, Yinchuan Ningxia)	VIN (100 µg/kg i.p., for seven consecutive days—700 µg/kg cumulative dose)	Mechanical allodynia (von Frey filaments), Thermal hyperalgesia and Cold allodynia (cold plate test)	15, 30 or 60 mg/kg, i.p	*Institute of Cancer Research (ICR) mice*	Pregabalin (10 mg/kg, i.p.)	**Mechanical allodynia:** Repeated but not single administration had effect on mechanical allodynia. **Cold allodynia:** Single and repeated administration suppressed cold allodynia **Thermal hyperalgesia:** No difference between control and treatment	Not reported	Dun et al. (2014)
Nicotine	Sigma-Aldrich (St. Louis, MO, United States)	PTX (8 mg/kg i.p., every other day for a total of four injections—32 mg/kg cumulative dose)	Mechanical allodynia (von Frey filaments)	Acute: 0.3, 0.6, or 0.9 mg/kg, i.p.; Chronic: 6, 12, or 24 mg/kg, s.c.	Male C57BL/6J mice	Not reported	**Acute:** reversed mechanical allodynia in paclitaxel-treated mice in a time- and dose-dependent **Chronic:** prevented the development of mechanical allodynia throughout the entire duration of the experiment, up to 35 days post-paclitaxel injection	Increased Acetylcholinesterase Activity	Kyte et al. (2018)
Nicotine	Not reported	OXL (2.4 mg/kg, i.p., 5 consecutive days every week for 3 weeks with a total of 15 injections—36 mg/kg cumulative dose)	Cold allodynia (cold plate test), Mechanical allodynia (paw pressure test) and Mechanical hyperalgesia (von Frey digital)	0.5, 1.0 or 1.5 mg/kg, i.p.	Male Sprague-Dawley rats	Not reported	The higher dose (1.5 mg/kg) was able to completely revert the cold allodynia, mechanical allodynia and mechanical hyperalgesia	Not reported	Di Cesare Mannelli et al. (2013)
Cannabinoids									
Cannabidiol	INSYS Therapeutics, Inc. (Austin, TX)	PTX (8 mg/kg, i.p., for 4 days—32 mg/kg cumulative dose), OXL (6 mg/kg, i.p. for 1 day) and VIN (0.1 mg/kg, i.p. for 7 days—0.7 mg/kg cumulative dose)	Mechanical allodynia (von Frey filaments)	0.0625—20 mg/kg, i.p. (PTX) 1.25—10 mg/kg, i.p. (OXL and VIN)	Male C57BL/6 mice	Not reported	Attenuation of mechanical sensitivity (PTX and OXL)	Not reported	King et al. (2017)
Cannabidiol	National Institute on Drug Abuse drug supply program (Bethesda, MD, United States)	PTX (8 mg/kg, i.p., on alternate days for 4 days—32 mg/kg cumulative dose)	Cold (acetone test) and Mechanical allodynia (von Frey filaments)	5 or 10 mg/kg, i.p	Male and Female C57BL/6 mice	Not reported	Prevented the development of paclitaxel-induced cold and mechanical allodynia	Not reported	Ward et al. (2011)
Cannabidiol	National Institute on Drug Abuse drug supply program (Bethesda, MD, United States)	PTX (8 mg/kg, i.p., on alternate days for 4 days—32 mg/kg cumulative dose)	Mechanical allodynia (von Frey filaments)	2.5, 5 or 10 mg/kg, i.p.	Female C57BL/6 mice	Not reported	Prevented paclitaxel-induced mechanical sensitivity.	5-HT1A receptors agonist	Ward et al. (2014)
Delta-9-tetrahydrocannabinol	National Institute on Drug Abuse drug supply program (Bethesda, MD, United States)	PTX (8 mg/kg, i.p., for 4 days—32 mg/kg cumulative dose), OXL (6 mg/kg, i.p. for 1 day) and VIN (0.1 mg/kg, i.p. for 7 days—0.7 mg/kg cumulative dose)	Mechanical allodynia (von Frey filaments)	0.0625—20 mg/kg, i.p. (PTX) 10 mg/kg, i.p. (OXL and VIN)	Male C57BL/6 mice	Not Reported	Attenuation of mechanical sensitivity (PTX and VIN)	Not Reported	King et al. (2017)
Curcuminoids									
Curcumin	Himedia Laboratories, (Mumbai, India)	VIN (0.1 mg/kg, i.p.—once a day for 7 days)	Thermal hyperalgesia (hot plate test), thermal allodynia (cold plate test), mechanical hyperalgesia (pin prick test)	15, 30 or 60 mg/kg, p.o.	Male Swiss mice	Pregabalin (10 mg/kg, p.o.)	Attenuated thermal hyperalgesia (30 and 60 mg/kg), thermal allodynia and mechanical hyperalgesia	Calcium inhibitory, and antioxidative activity (increased SOD, CAT, GPx and GSH, and decreased LPO and NO)	Babu et al. (2015)
Tetrahydrocurcumin	Sami labs (Bangalore)	VIN (75 µg/kg, i.p., once per day for 10 consecutive days—750 µg/kg cumulative dose)	Thermal hyperalgesia (hot plate test), thermal allodynia (cold plate test) and mechanical hyperalgesia (Randall and Sellito test)	40 or 80 mg/kg, p.o.	Male Wistar rats	Pregabalin (10 mg/kg, p.o.)	Increased the pain threshold	Decreased TNF-α, calcium inhibitory and antioxidant activity (Increased CAT, SOD, GPx, GSH and decreased LPO, NO)	Greeshma et al. (2015)
Flavonoids									
6-Methoxyflavanone	Sigma-Aldrich, (United States)	CIS (3 mg/kg, i.p./week for 4 weeks—12 mg/kg cumulative dose)	Mechanical Allodynia [static (von Frey filaments) and dynamic (cotton bud pressure)]	25, 50, or 75 mg/kg, i.p.	Sprague-Dawley rats	Gapapentine (75 mg/kg, i.p)	Reduced the cisplatin-induced static allodynia and increased the dynamic allodynia through increasing the paw withdrawal latency	COX-1 and 2 antagonists (*in vitro*)	Akbar et al. (2020)
6-Methoxyflavone	Sigma-Aldrich (St. Louis, MO, United States)	CIS (3.0 mg/kg, i.p. once a week for 4 weeks—12 mg/kg cumulative dose)	Mechanical allodynia (abdominal constriction test) and Thermal hyperalgesia (hot plate and tail immersion tests)	25, 50 or 75 mg/kg, i.p.	Male Sprague-Dawley rats	Gabapentine (75 mg/kg, i.p.)	Increased the paw withdrawal threshold and protected against temporal expression of hypoalgesia	Not reported	Shahid et al. (2017)
Icariin	Shanghai CIVI Chemical Technology Co. (China)	PTX (8 mg/kg, i.p., for 3 days—24 mg/kg cumulative dose)	Mechanical allodynia (von Frey digital)	25, 50 or 100 mg/kg, i.p.	Male Sprague-Dawley rats	Not reported	Alleviated paclitaxel-induced mechanical allodynia in the long term (100 mg/kg)	Decreased expression of TNF-a, IL-1b and IL-6, NF-kB and GFAP, SIRT1 downregulation and H4 acetylation in the spinal cord (100 mg/kg)	Gui et al. (2018)
Quercetin	Not reported	OXL (1 mg/kg, i.v., twice a week with a total of nine injections—9 mg/kg cumulative dose)	Mechanical hyperalgesia (von Frey digital) and Cold allodynia (tail immersion test)	25, 50 or 100 mg/kg, i.p.	Male Swiss mice	Not reported	Increased mechanical and cold nociceptive threshold	Prevention of lipid peroxidation and tyrosine nitrosylation, Decreased iNOS expression	Azevedo et al. (2013)
Quercetin	Not reported	PTX (2 mg/kg, i.p., every other day with a total of 4 injections—8 mg/kg cumulative dose)	Mechanical allodynia (von Frey filaments) and Thermal hyperalgesia (tail immersion test)	20 or 60 mg/kg, intragastrically	Male Sprague-Dawley rats and Institute of Cancer Research (ICR) mice	Not reported	Reduced mechanical nociceptive threshold and increased tail withdrawal latency	Decreased levels of proteins PKCε and expression of TRPV1	Gao et al. (2016)
Rutin	Not reported	OXL (1 mg/kg, i.v., twice a week with a total of nine injections—9 mg/kg cumulative dose)	Mechanical hyperalgesia (von Frey digital) and Cold allodynia (tail immersion test)	25, 50 or 100 mg/kg, i.p.	Male Swiss mice	Not reported	Increased mechanical and cold nociceptive threshold	Prevention of lipid peroxidation and tyrosine nitrosylation, Decreased iNOS expression	Azevedo et al. (2013)
Terpenes									
Aucubin	Wako Pure Chemical Industries (Osaka, Japan)	PTX (5 mg/kg, i.p.—single administration)	Mechanical Allodynia (von Frey filaments)	5, 15 or 50 mg/kg, i.p.	Male C57BL/6NCr mice	Not reported	Significantly inhibited the exacerbation of allodynia (15 and 50 mg/kg)	Not reported	Andoh et al. (2016)
Aucubin	Wako Pure Chemical Industries (Osaka, Japan)	PTX (5 mg/kg, i.p.—single administration)	Mechanical Allodynia (von Frey filaments)	50 mg/kg, i.p.	Male C57BL/6NCr mice	Not reported	Inhibited exacerbation of paclitaxel-induced mechanical allodynia	Inhibition of Endoplasmic Reticulum (CCAAT/enhancer-binding protein) ER Stress (inhibited CHOP expression) Schwann Cells	Andoh et al. (2017)
Betulinic acid	Extracted and isolated in *Hyptis emoryi*	PTX (2 mg/kg, i.p., every other day with a total of 4 injections—8 mg/kg cumulative dose)	Mechanical allodynia (von Frey filaments)	2 µg/5 µl, i.th.	Male and female Sprague-Dawley rats	Not reported	Significant reversal of mechanical allodynia at 1 h after injection, lasting for 2 additional hours	Block of N- and T-type calcium channels	Bellampalli et al. (2019)
(−)-Hardwickiic Acid	Isolated in *Croton calcifornicus*	PTX (2 mg/kg, i.p., every other day with a total of 4 injections—8 mg/kg cumulative dose)	Mechanical allodynia (von Frey filaments)	2 µg/5 µl, i.th.	Male and female Sprague-Dawley rats	Not reported	Significant relief of mechanical allodynia	Blockade of Tetrodotoxin-Sensitive Voltage-Dependent Sodium Channels	Cai et al. (2019)
Hautriwaic Acid	Isolated in *Eremocarpus setigerus*	PTX (2 mg/kg, i.p., every other day with a total of 4 injections—8 mg/kg cumulative dose)	Mechanical allodynia (von Frey filaments)	2 µg/5 µl, i.th.	Male and female Sprague-Dawley rats	Not reported	No reversal of mechanical allodynia	Blockade of Tetrodotoxin-Sensitive Voltage-Dependent Sodium Channels	Cai et al. (2019)
Pedicularis-lactone	Extraction and isolation in *Viticis Fructus*	PTX (5 mg/kg, i.p., single injection)	Mechanical allodynia (von Frey filaments)	15 mg/kg p.o. (considering purity 10.1 mg/kg)	Male C57BL/6NCr mice	Not reported	Inhibited PTX-induced allodynia	Not reported	Yu et al. (2021)
Viteoid I	Extraction and isolation in *Viticis Fructus*	PTX (5 mg/kg, i.p., single injection)	Mechanical allodynia (von Frey filaments)	15 mg/kg p.o. (considering purity 13.8 mg/kg)	Male C57BL/6NCr mice	Not reported	Inhibited PTX-induced allodynia	Not reported	Yu et al. (2021)
Viteoid II	Extraction and isolation in *Viticis Fructus*	PTX (5 mg/kg, i.p., single injection)	Mechanical allodynia (von Frey filaments)	15 mg/kg p.o. (considering purity 13.0 mg/kg)	Male C57BL/6NCr mice	Not reported	Inhibited PTX-induced allodynia	Not reported	Yu et al. (2021)
Xanthines									
Propentofylline	Not reported	VIN (75 µg/kg i.v. 5 injections daily, 3 days of interval then 4 more doses, total 9 injections—675 µg/kg cumulative dose)	Mechanical allodynia (von Frey filaments)	10 mg/kg, i.p.	Male Holtzman rats	Not reported	Treatment inhibited any mechanical allodynia	Glial modulation	Sweitzer et al. (2006)

PTX: paclitaxel; CIS: cisplatin; OXL: oxaliplatin; VIN: vincristine.

The compounds used for the studies were obtained mostly by purchase in laboratories and specialized companies (*n* = 15, 57.70%, Table 1). Other studies extracted and isolated the compound from plant species (*n* = 6, 23.07%), and some did not report the obtaining methods (*n* = 5, 19.23%). Although the techniques for isolating and characterizing new compounds from medicinal plants have evolved over the years, some challenges are still encountered. Among them, the following stand out: the need for multiple separation techniques to isolate the target compound, the low yield of purified compounds, the influence of environmental factors on the production of secondary metabolites, in addition to the high cost and time (Sasidharan et al., 2010; Atanasov et al., 2015). Thus, it is believed that this is the reason why pharmacology research groups have opted for the commercial acquisition of the compounds tested.

Preclinical studies found were developed in mice (*n* = 14, 56.00%), rats (*n* = 10, 40.00%) or both (*n* = 1, 4.00%), with variation in the strain and sex of the animal used in each study. For the rats, the strains used were Sprague-Dawley, Wistar and Holtzman, whereas the mouse strains used were C57BL/6, C57BL/6J, C57BL/6NCr, Swiss and Institute of Cancer Research (ICR). Among rodents, mice have in fact been more used in preclinical studies of this nature, as they are easier to handle, cheaper and are required in smaller quantities. However, the results must be evaluated with caution, since the nociceptive response tends to vary between different species and sex (Le Bars et al., 2001; Smith, 2019).

For induction of peripheral neuropathy, four types of chemotherapy were found as inducers: paclitaxel (PTX) (*n* = 16, 53.33%), vincristine (VIN) (*n* = 6, 20.00%), oxaliplatin (OXL) (*n* = 6, 20.00%) and cisplatin (*n* = 2, 6.67%). The protocols used for induction differ in several aspects, even for the same chemotherapeutic agent, which may vary among themselves in frequency, dose and route of administration, besides cumulative dose (Table 1). In literature, most chemotherapeutic substances that cause peripheral neuropathy in cancer patients have been used to develop CIPN models in rodents. Among the most used are platinum compounds (cisplatin, carboplatin and oxaliplatin), taxanes (paclitaxel, docetaxel), proteasome inhibitors bortezomib, carfilzomib, vinca alkaloids (vincristine) and others, including thalidomide and suramin (Höke and Ray, 2014). These compounds act in several pathways inducing CIPN (Eldridge et al., 2020), as discussed below in this article. However, the lack of standardization of the models regarding the inducing agent and animals used corresponds to an important disparity factor (Höke, 2012; Höke and Ray, 2014) and is still a challenge for this field of research, making it impossible to carry out quantitative analysis in secondary studies that aim to demonstrate evidence of these findings together.

The main methods used by all studies to evaluate the compound for its nociceptive activity were: Mechanical Allodynia (*n* = 21), Thermal Allodynia (*n* = 8), Thermal Hyperalgesia (*n* = 8) and Mechanical Hyperalgesia (*n* = 5), where each study used one or more simultaneous techniques for evaluation. These nociceptive responses involve different painful conduction pathways and mimic well the symptoms experienced by patients with CIPN. In allodynia, non-noxious stimuli induce nociception due to increased sensitivity of Aβ fibers, unlike hyperalgesia, in which noxious stimuli induce a greater nociceptive response. This mechanism is associated with hypersensitization of Aδ fibers (Sandkühler, 2009).

Besides, some studies have used standard drugs as a positive control, such as Pregabalin (*n* = 3), Gabapentin (*n* = 2) and Amitriptyline (*n* = 1), but most have not used or reported the use of any type of substance as positive control (*n* = 21). The low use of drugs known as positive control is justified by the lack of suitable pharmacotherapy for the treatment of this painful condition. Thus, antidepressants and anticonvulsants have been used clinical strategies, as they modulate neutral transmission systems, helping to control symptoms associated with CPIN (Hou et al., 2018).

### 3.2 Description of the plant-based natural products used

#### 3.2.1 Terpenes

Terpenes are the largest class of natural products, having more than 55,000 compounds known and diversified by chemical structure. They are compounds found in several organisms, both animal and plant (Bergman et al., 2019). A range of pharmacological activities are studied and described in the literature involving compounds of this class, in particular, the analgesic effect (Guimarães et al., 2014; Gouveia et al., 2018).

Aucubin (C_15_H_22_O_9_) is one of the main components of *Plantaginis semen*, a plant that makes up traditional Chinese medicine Goshajinkigan (Samuelsen, 2000), which is known to attenuate the progression of peripheral neuropathy induced by treatment with docetaxel and PTX/carboplatin in cancer patients (Andoh et al., 2016). In this same study, Andoh et al. (2016), used a model of peripheral neuropathy induced by PTX in mice, and aucubin, in the highest tested doses (15 and 50 mg/kg, i.p.), significantly inhibited the exacerbation of mechanical allodynia. Another study used this compound in the largest dose (50 mg/kg, i.p.) and found a similar antiallodynic effect, showing that aucubin inhibits the endoplasmic reticulum resulting in the inhibition of the oxidative stress marker (CHOP) and decreasing damage to Schwann cells (Andoh et al., 2017).

Pedicularis-lactone is a glycosidic iridoid belonging to the class of monoterpenes extracted and isolated from the *Viticis rotundifolia* L. plant (Chaudhry et al., 2020). In a test of mechanical allodynia in the model of peripheral neuropathy induced by PTX, it was reported that the compound in the dose used (15 mg/kg p.o.) inhibited allodynia caused by the chemotherapy in mice (Yu et al., 2021).

Viteoid I (C_9_H_12_O_4_), belonging to the monoterperne class because it is a glycosidic iridoid, isolated and extracted from the *Viticis Fructus* plant, presented an antiallodynamic effect in a model of peripheral neuropathy induced by PTX in mice, using a dose of 15 mg/kg (p.o.) (Yu et al., 2021).

Viteoid II (C_9_H_12_O_4_) is a glycosidic iridoid, monoterpene, isolated and extracted from the *Vicitis Fructus* plant (Ono et al., 1997). It was tested in a chemotherapeutic-induced peripheral neuropathy (PTX) model, with the effect of reversing the mechanical allodynia felt by the animals at the dose tested (15 mg/kg, p.o.) (Yu et al., 2021).

(−)-Hardwickiic acid (C_20_H_28_O_3_) is a diterpene isolated and extracted from the *Croton calcifornicus* plant with potential analgesic effect (Crentsil et al., 2020). The compound was effective in significantly relieving the mechanical allodynia of rats induced to peripheral neuropathy with PTX at the dose tested (2 µg/5 µl, i.th.). In fact, it may be linked to the fact that the compound blocks voltage-dependent sodium channels sensitive to tetrodotoxin (Cai et al., 2019).

Hautriwaic acid (C_20_H_28_O_4_) is a diterpene isolated from the plant *Eremocarpus setigerus* with anti-inflammatory activity already described in the literature (Jolad et al., 1982; Salinas-Sánchez et al., 2012). It was tested in a model of PTX-induced peripheral neuropathy for its antiallodynic effect in rats. In contrast, at the dose used (2 µg/5 µl, i.th.), the substance was not able to reverse the mechanical sensitivity of the animals, despite having the same ability to block voltage-sensitive sodium channels dependent on tetrodotoxin from the previous compound (Cai et al., 2019).

Betulinic acid (C_30_H_48_O_3_) is a triterpene isolated from the plant *Hyptis emoryi*. A number of biological effects are described by this compound, including antitumor, anti-inflammatory and antiviral (Ríos and Máñez, 2018). Evaluating its effect in a model of PTX-induced peripheral neuropathy, betulinic acid was able to significantly reverse mechanical allodynia at the dose tested (2 µg/5 µl, i.th.), highlighting the ability to block calcium-type channels N and T (Bellampalli et al., 2019).

#### 3.2.2 Alkaloids

Identified predominantly in plants, the alkaloids form a large group of secondary metabolites, presenting more than 12,000 different structures and a high diversity, with the biological effects on mammals being explored since remote times (Schläger and Dräger, 2016).

Berberine (C_20_H_18_NO_4_+) is a quaternary benzoisoquinolic alkaloid, derived from the Berberis species and used in Ayurvedic and Chinese medicines for the treatment of various diseases (Mokhber-Dezfuli et al., 2014). The administration of PTX developed neuropathy in the treated rats that was shown by the significant reduction of the animals’ latency time in the tail flick test for thermal hyperalgesia and in the cold allodynia test. Also, the treatment with berberine in the doses of 10 and 20 mg/kg i.p. indicated a neuroprotective effect in both tests, inhibiting the decrease in latency caused by PTX, an effect similar to the positive control used (amitriptyline 10 mg/kg, i.p.). The study demonstrated an increase in the expression of the Nrf2 gene, responsible for antioxidant defense enzymes, in addition to the antioxidant activity of the compound by decreasing TBARS levels and raising SOD, GPx, and GSH levels, all markers of oxidative stress (Singh et al., 2019).

Evodiamine (C_19_H_17_N_3O_) is the major compound isolated from *Evodia ruaecarpa,* characterized as a quinolone alkaloid. It has been used in traditional Chinese medicine for the treatment of headaches, postpartum hemorrhage and gastrointestinal disorders (Jiang and Hu, 2009). The injection of PTX significantly reduced the withdrawal threshold of the rat’s paw, indicating that neuropathy was induced in the animals, being inhibited by the administration of evodiamine at a dose of 5 mg/kg i.p. both in the mechanical allodynia test by von Frey filaments and in the hot plate test, which assesses thermal hyperalgesia. Treatment with evodiamine also reduced the number of suppressed intraepidermal nerve fibers, one of the mechanisms of PTX to cause peripheral neuropathy, in addition to inhibiting the increase in inflammatory cytokines (IL-1β, IL-6, TNF-α, and MCP-1), inhibition of oxidative stress (MDA, NO, and 8-isoprostane F2α) and maintenance of mitochondrial function (inhibition of MMP reduction and expression of PGC-1α, UCP2, and SOD2 proteins) (Wu and Chen, 2019).

Levo-tetrahydropalmatine (C_21_H_25_NO_4_) is one of the major components of *Corydalis yanhusuo*, an herb used for more than 40 years in traditional Chinese medicine as an analgesic with sedative and hypnotic properties, and the isolated substance used to treat headaches and other moderate pains (Ma et al., 2008; Lee et al., 2014; Zhou et al., 2016). Chronic treatment with oxaliplatin induced mechanical hyperalgesia in the mice, indicated by the decrease in the paw withdrawal threshold by the animals, a behavior that was inhibited by the administration of levo-tetrahydropalmatine in a dose-dependent manner, where the highest dose used (4 mg/kg; i.p.) managed to increase threshold of paw withdrawal to levels prior to the induction of neuropathy. In order to try to understand the mechanism involved in the effect of the compound, a dopaminergic D1 receptors antagonist was used, significantly attenuating the anti-hyperalgesic effect of levo-tetrahydropalmatine (Guo et al., 2014).

Matrine (C_15_H_24_N_2_O) is a quaternary quinolizidine alkaloid extracted from the traditional Chinese herb *Sophora flavecens* Ait. (*Kushen*), being used commonly as antiviral, anti-fibrotic, anti-inflammatory, anti-arrhythmic, anti-anaphylactic, and immunosuppressive (Liu et al., 2003; Huang and Xu, 2016; You et al., 2020; Li et al., 2021). In this study, matrine was evaluated for the antinociceptive effect caused by the peripheral neuropathy induced by VIN. In the evaluation of mechanical allodynia, a single dose of matrine (60 mg/kg, i.p.) was not able to mitigate the effects of allodynia caused by VIN. Nonetheless, the daily administration at all doses (15, 30, and 60 mg/kg) significantly reversed the mechanical and thermal allodynia caused by the chemotherapy, an effect similar to the positive control used (pregabalin 10 mg/kg, i.p.). However, the compound showed no difference for the control in the hot plate test that evaluates thermal hyperalgesia (Dun et al., 2014).

Nicotine (C_10_H_14_N_2_) is a polycyclic alkaloid present in the leaves of the species *Nicotiana tabacum*. It has been demonstrating a potential utility as an analgesic, antinociceptive and anti-inflammatory in preclinical and clinical studies (Kyte et al., 2018), being justified by growing interest in nicotinic receptors for pain relief (Di Cesare Mannelli et al., 2013). The effect of nicotine was evaluated for its activity against cold allodynia, mechanical allodynia and mechanical hyperalgesia in a model of peripheral neuropathy induced by OXL in rats, where the highest dose (1.5 mg/kg, ip) was able to completely reverse all effects caused by chemotherapy (Di Cesare Mannelli et al., 2013). This compound was also tested in a PTX-induced peripheral neuropathy model in mice, where both acute and chronic mechanical allodynia were investigated. Nicotine reduced acute allodynia at all doses tested (0.3, 0.6, and 0.9 mg/kg, ip) and prevented chronic allodynia (6, 12, and 24 mg/kg, s.c.) for 35 days after the PTX injection. It has also been suggested that nicotine is acting through nicotinic acetylcholine receptors, so that the effect of nicotine has been effectively blocked by an antagonist of these channels (Kyte et al., 2018).

#### 3.2.3 Flavonoids

Flavonoids belong to a group of natural substances with variable phenolic structure and are found in different parts of plants. They are known for their beneficial health effects. More than 4,000 varieties of flavonoids have been identified and these can be divided into several classes based on their molecular structure (Nijveldt et al., 2001).

6-Methoxyflavanone (C_16_H_14_O_3_) is a positive allosteric modulator of GABA responses in recombinant human GABA_A_ receptors, becoming a target of studies for clinical conditions involving this type of action (Hall et al., 2014). In a CIS-induced peripheral neuropathy model, this compound was able to reduce static allodynia, in addition to increasing the paw withdrawal latency time in the dynamic allodynia test caused by chemotherapy (50, 75 mg/kg, i.p.). Also, the highest dose (75 mg/kg) obtained similar results when compared to gabapentin (75 mg/kg, i.p.), the positive control used. It was also seen that 6-methoxyflavanone has a strong antagonistic tendency to both COX-1 and COX-2 enzymes, in order to help elucidate its mechanisms of action (Akbar et al., 2020).

6-Methoxyflavone (C_16_H_12_O_3_) also acts as a positive modulator of GABA responses in recombinant human GABA_A_ receptors, in addition to having immunomodulatory activities, being the focus of study for its antinociceptive activity (Hall et al., 2014). At all doses tested (25, 50, and 75 mg/kg, i.p.), this compound was able to increase the paw withdrawal threshold in a model of mechanical allodynia and thermal hyperalgesia induced by CIS in rats, having an effect similar to the positive control used (gabapentin 75 mg/kg, i.p.) (Shahid et al., 2017).

Icariin (C_33_H_40_O_15_) is a flavonoid extracted from *Epidemium brevicornum* Maxim, with activities already known in the literature as anti-inflammatory and anticancer (Zhang et al., 2008; He et al., 2020). Using a model of peripheral neuropathy caused by PTX in rats, it was seen that this compound was able to relieve mechanical allodynia caused by chemotherapy in the long term, when using the highest dose (100 mg/kg, i.p.). It has been suggested that icariin exerts its antinociceptive effects by decreasing the expression of inflammatory mediators (TNF-α, IL-1β, and IL-6), by the negative regulation of oxidative stress markers (NF-κB, GFAP, and SIRT1), in addition to performing H4 acetylation in the spinal cord (Gui et al., 2018).

Quercetin (C_15_H_10_O_7_) is a polyphenolic flavonoid found in several plants. It has activities well described in the literature as antioxidant (Song et al., 2020), anti-inflammatory (Carullo et al., 2017) and anticarcinogenic (Reyes-Farias and Carrasco-Pozo, 2019). There are studies in the literature about its antinociceptive activity in a model of peripheral neuropathy caused by chemotherapy (Azevedo et al., 2013). In an OXL model in mice, quercetin at all doses (25, 50, and 100 mg/kg, i.p.) increased the nociceptive threshold in both mechanical hyperalgesia and thermal allodynia (Azevedo et al., 2013). In a PTX model, evaluating mechanical allodynia and thermal hyperalgesia, this compound (20 and 60 mg/kg, intragastric) provided a reduction in the mechanical nociception threshold and increased tail withdrawal latency (Gao et al., 2016). Some mechanisms have already been proposed to justify the action of quercetin, due to its antioxidant activity (prevention of lipid peroxidation, tyrosine nitrosylation and decreased iNOS expression), and by decreasing the levels of PKCε proteins and TRPV1 expression (Azevedo et al., 2013; Gao et al., 2016).

Rutin (C_27_H_30_O_16_) is a bioflavonoid with pronounced antioxidant activity. It is water-soluble and is converted to quercetin in the bloodstream, with several studies elucidating its anti-inflammatory (Arowoogun et al., 2021), antioxidant (Enogieru et al., 2018; Saleh et al., 2019) and analgesic activities (Alonso-Castro et al., 2017). Evaluating its effect on peripheral neuropathy caused by OXL, it had a significant effect on all doses (25, 50, and 100 mg/kg, i.p.) in the tests of mechanical hyperalgesia. It also increased the nociceptive threshold of thermal allodynia at the two highest doses (50 and 100 mg/kg, i.p.). It has been reported that rutin is capable of preventing lipid peroxidation and tyrosine nitrosylation, in addition to decreasing iNOS expression, strengthening the findings and their antioxidant activity (Azevedo et al., 2013).

#### 3.2.4 Cannabinoids

Term initially used to designate a specific group of compounds present in the *Cannabis sativa* plant, popularly known for its psychoactive activity, it has been used since ancient times, particularly in traditional Chinese medicine for asthma, malaria and gout, and in India for neuralgia, seizures and migraines. Since the discovery of specific receptors for these compounds, there has been a growing public and clinical interest in using them for the management of diseases and symptoms (Fraguas-Sánchez and Torres-Suárez, 2018).

Cannabidiol (C_21_H_30_O_2_) is a phytocannabinoid that has been gaining attention for the treatment of neuropathic pain due to its high affinity for cannabinoid receptors and consequently its side effects, while exercising anti-inflammatory and analgesic activities (Burstein, 2015; Britch et al., 2021). Studies on the activity of this compound in chemotherapy-induced peripheral neuropathy have already evaluated its antinociceptive effect against the causative agents PTX, OXL, and VIN. In a model of PTX-induced peripheral neuropathy in mice, this substance was able to prevent the development of mechanical and thermal sensitivity in varying doses (2.5, 5, and 10 mg/kg, ip) (Ward et al., 2011; Ward et al., 2014). This characteristic has been completely mitigated by the administration of a 5-HT_1A_ receptor antagonist suggesting that the biological activity of cannabidiol is linked serotonergic receptors (Ward et al., 2014). It was also shown that this compound was able to attenuate the mechanical sensitivity in the peripheral neuropathy model induced by OXL, but not by VIN (King et al., 2017).

Delta-9-tetrahydrocannabinol (C_21_H_30_O_2_) is one of the main exogenous cannabinoid compounds and is found as a major component in most plants of the *Cannabis* family. It acts as a mixed agonist of both CB1 and CB2 receptors (Pertwee, 2008). It was studied whether delta-9-tetrahydrocannabinol was potent and effective against peripheral neuropathy induced by PTX, OXL and VIN in mice (King et al., 2017). In the test of mechanical allodynia using von Frey filaments, the compound was able to attenuate the dose-related PTX-induced peripheral neuropathy, with significant effect in the doses of 2.5 and 10 mg/kg (i.p.). Using a dose of 10 mg/kg (i.p.), it was seen that this cannabinoid was able to attenuate the mechanical sensitivity of the mice in the VIN induction model, but not by OXL (King et al., 2017).

#### 3.2.5 Curcuminoids

Compounds derived from curcumin, a major component of *Curcuma longa* L., known in traditional Chinese medicine as turmeric, were first extracted in the 19th century, and in the past two decades they have received great attention due to their biofunctional activities, such as anti-tumor, antioxidant and anti-inflammatory. These activities are attributed to the elements present in the molecular structure of curcuminoids (Tomeh et al., 2019).

Curcumin (C_21_H_20_O_6_) is the main representative of the class. It has been shown to be non-toxic and non-mutagenic, with a wide range of biological activities. Studies of its antinociceptive activity in different animal models of pain have been published (Chen et al., 2015; Xiao et al., 2017). Its effect in a model of VIN-induced neuropathy was evaluated against mechanical and thermal hyperalgesia and thermal allodynia, using doses of 15, 30, and 60 mg/kg (i.p.). It was seen that in the two highest doses, curcumin was able to attenuate the mechanical and thermal sensitivity caused by chemotherapy, an effect similar to that presented by the positive control used (pregabalin 10 mg/kg, i.p.) (Babu et al., 2015).

Tetrahydrocurcumin (C_21_H_24_O_6_) is a metabolite of curcumin, believed to be more bioavailable and potent than its precursor due to its more hydrogen molecules. It has activities already reported in the literature as more potent than curcumin such as anti-inflammatory and antioxidant (Pan et al., 2020). In a model of VIN-induced peripheral neuropathy, this compound (40 and 80 mg/kg, p.o.) was able to increase the nociceptive threshold of rats for thermal and mechanical hyperalgesia, in addition to thermal allodynia, an effect similar to that presented by the positive control employed in tests (pregabalin, 10 mg/kg, p.o.). Some mechanisms may be involved in the effect exerted by tetrahydrocurcumin to attenuate peripheral neuropathy caused by chemotherapy, including its ability to reduce inflammatory cytokine TNF-α, decrease in total calcium levels, and by its antioxidant activity (increased levels of CAT, SOD, GPx, GSH, and decreased LPO and NO) (Greeshma et al., 2015).

#### 3.2.6 Xanthines

Propentophylline (C_15_H_22_N_4_O_3_) is a methylxanthine that has the ability to modulate microglia cells and inhibit the production of free radicals, with studies that describe its profound neuroprotective, antiproliferative and anti-inflammatory activity (Meiners et al., 2004). In the evaluation of mechanical allodynia induced by VIN, this compound (10 mg/kg; i.p.) was able to inhibit any mechanical allodynia that the rat might present, an effect possibly accomplished by the ability of glial modulation presented by the compound (Sweitzer et al., 2006).

### 3.3 Mechanism of action

CIPN presents complex pathophysiology, which varies between the different groups of chemotherapy agents (Zajączkowska et al., 2019). Although the mechanisms behind CIPN induction remain uncertain and controversial, it is generally known that neurotoxic chemotherapeutic compounds cause irreversible damage to peripheral nerves, an effect caused by deterioration to microtubules, directly interfering with axonal transport, as well as causing neuronal cell death by DNA cytotoxic effects (Chine et al., 2019). When there is damage to the myelin sheath, there is also CIPN generation; this mechanism is presented by taxanes, vinca alkaloids and proteosome inhibitors. The terpene aucubin showed a decrease in the oxidative stress of the endoplasmic reticulum, leading to a protection of Schwann cells, thus preserving the myelin sheath of the axons, reducing the mechanical allodynia presented by mice (Andoh et al., 2017).

Oxidative stress has been shown to be one of the main pathogenic factors that contribute to damage to peripheral sensory neurons. Several inducing agents lead to oxidative stress and mitochondrial damage, such as platinum compounds, vinca alkaloids, taxanes and proteosome inhibitors, already proven in the literature that reducing oxidative stress is a promising therapy in the treatment of CIPN (Shim et al., 2019). This review showed several natural products from plants that showed antioxidant action, reducing substances that promote oxidative stress, as well as increasing the activity of the antioxidant enzymes CAT, GPx, GSH, and SOD (Azevedo et al., 2013; Babu et al., 2015; Greeshma et al., 2015; Andoh et al., 2017; Gui et al., 2018; Singh et al., 2019; Wu and Chen, 2019), highlighting the potential of these compounds in attenuating CIPN.

Besides, neuroinflammation is involved in several aspects of peripheral neuropathy models in general, and its action on CIPN is evidenced when chemotherapy substances are able to penetrate the blood-brain barrier, binding and accumulating in dorsal root ganglia and peripheral axons. In the activation of immune glial cells, these drugs cause the secretion of mediators that increase neuronal excitability and generate hypersensitivity to pain. Neuro-inflammation occurs in the spinal cord, associated with astrocyte activation, without microglial involvement (Makker et al., 2017). In this context, several natural products cited in this review reported anti-inflammatory action by reducing pro-inflammatory cytokines such as TNF-α, IL-1β, MCP-1, and IL-6, for example, the alkaloid evodiamine (Wu and Chen, 2019), the tetrahydrocurcumin (Babu et al., 2015), the flavonoid icariin (Gui et al., 2018), in addition to showing activity in glial modulation such as the xanthine propentophylline (Sweitzer et al., 2006).

An alteration in the expression and function of ion channels is another mechanism that contributes to the development of CIPN. As shown by Zajączkowska et al. (2019), the decreased expression of potassium channels (K^+^) leads to a spontaneous activation of nociceptors observed in dorsal root ganglia. Furthermore, the activation of sodium channels (Na^+^), besides TRPV1 and TRPA1, was observed in root ganglia neurons dorsal in CIPN models, being important components in the pain signaling pathway. The alkaloid (−)-Hardwickiic Acid (Cai et al., 2019) showed the ability to block voltage-dependent sodium channels sensitive to tetrodotoxin, which may be one of the mechanisms responsible for the reduction of mechanical allodynia. Similarly, the flavonoid quercetin was able to reduce the activation of the TRPV1 cationic channels, helping to reduce the mechanical nociception caused by PTX (Gao et al., 2016).

Finally, changes in Ca^2+^ homeostasis are known to participate in the pathogenesis of CIPN, with an intracellular dysregulation of calcium caused by the chemotherapeutic substance being observed, leading to an increased expression in the activity of calcium channels in experimental animals (Zajączkowska et al., 2019). Some compounds presented in this review such as curcumin (Babu et al., 2015) and tetrahydrocurcumin (Greeshma et al., 2015) showed calcium inhibitory activity, and the terpene betulinic acid was able to block N and T-type calcium channels, already known to directly participate in the control of neuropathies (Bellampalli et al., 2019).

Thus, the compounds tested seem to demonstrate a high potential for the control of CIPN through the modulation of several model targets involved in the genesis and maintenance of this challenging painful condition.

### 3.4 Toxicity

The concern with determining the safety of drugs approved for use is one of the main themes of several amendments, laws or acts elaborated in recent years, often undergoing adjustments to ensure a positive risk-benefit ratio for users (Alshammari, 2016). The fact that CIPN causes a significant loss of functional abilities and negatively affects quality of life can lead to dose reductions, discontinuation of treatment and, ultimately, affect the overall survival of patients (Hou et al., 2018). That demonstrates the need that compounds for the treatment of these conditions are safe and do not cause further discomfort or obstacles to patients.

Some compounds represented in this review have previous studies that determine the lethal dose 50 (LD_50_) by acute assessment. Nicotine was shown to have an LD_50_ of 33.5 mg/kg in rats after subcutaneous administration of the substance (King and Becker, 1966). Evodiamine in an acute toxicity model in rats obtained an LD_50_ of 77.79 mg/kg, i.v., with no adverse effects being observed, in addition to weight loss (Yang et al., 2006). One study determined the LD_50_ of delta-9-tetrahydrocannabinol by various routes of administration, reporting an LD_50_ of 800–1,270 mg/kg intragastrically and 36–40 mg/kg by intravenous and inhaled administration (Rosenkrantz et al., 1974). In turn, cannabidiol presented an LD_50_ of 212 mg/kg, i.v. in rhesus monkeys for 9 days of administration, where tremors and inhibition of the central nervous system (depression, sedation and prostration) were presented (Rosenkrantz et al., 1981). The administration of rutin in rats and guinea pigs at a dose of 30–50 mg/kg intravenously and intraperitoneally showed no signs of acute or chronic toxicity (Wilson et al., 1947). Matrine presented an LD_50_ of 157.13 mg/kg, i.p. in Kunming mice, with degenerative changes in nerve cells in brain tissue being observed, suggesting a tolerable dose of 80 mg/kg (Wang et al., 2010).

Besides, previous studies of some other substances, despite not determining the LD_50_, report aspects about safety or adverse effects presented. It is reported in the literature that compounds such as aucubin (Zeng et al., 2020), betulinic acid (Mullauer et al., 2010), quercetin (Pal and Tripathi, 2020), tetrahydrocurcumin (Majeed et al., 2019) and tetrahydropalmatine (Wang et al., 2017) did not show significant changes or serious adverse reactions when administered to rodents. Highlights for curcumin, which has been shown to be safe for use in six different clinical studies with humans (Chainani-Wu, 2003).

Although some preclinical studies have already addressed the issue of toxicity of various natural products from plants, information about the maximum allowed dose, drug interactions and adverse effects is very scarce, making more studies with this focus necessary.

### 3.5 Risk of bias and quality of selected studies

**FIGURE 3:**
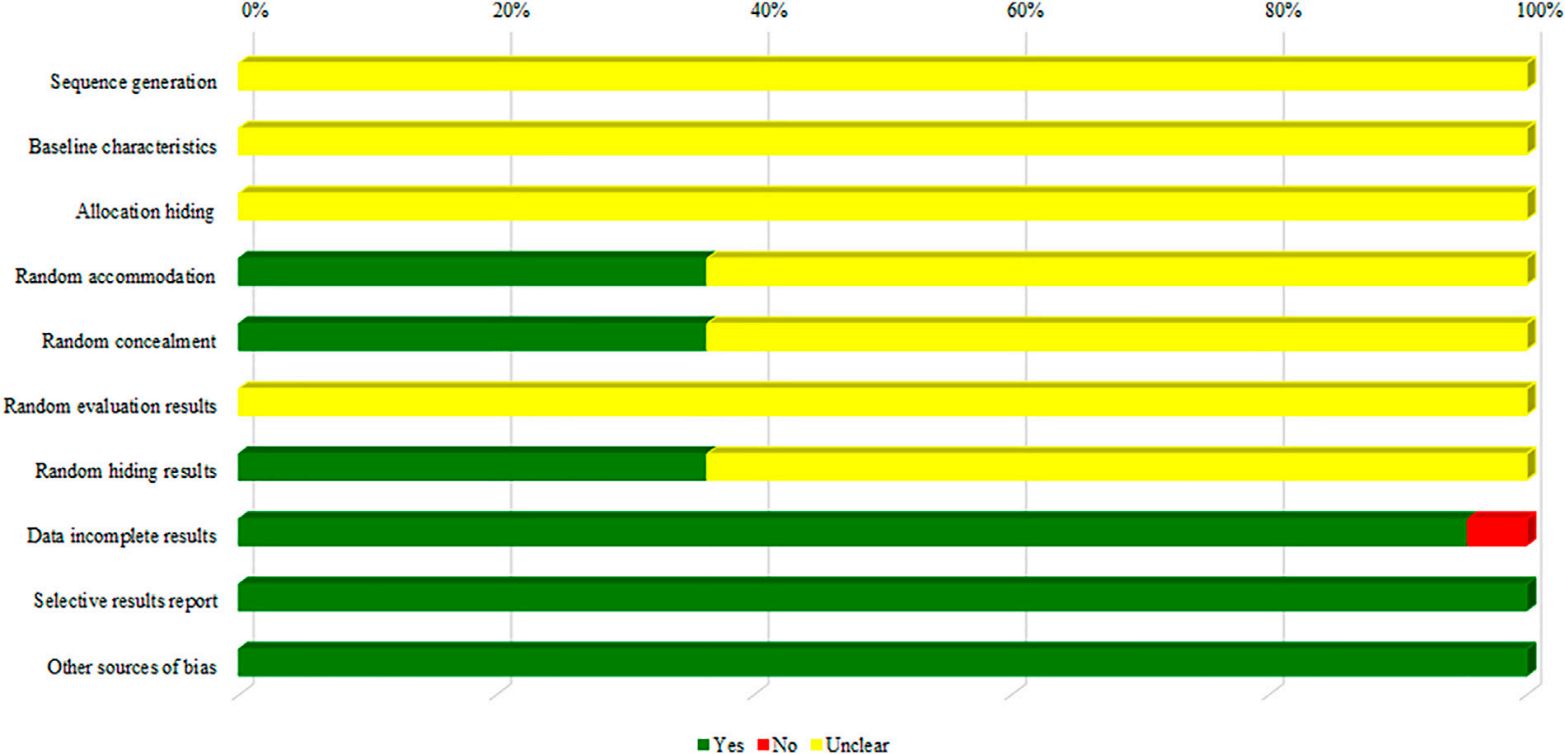
Risk-of-bias assessment of the preclinical trials included in this systematic review.

Most of the studies selected when evaluated by the SYRCLE tool for methodological quality and risk of bias could not be evaluated in several determined fields due to the lack of information about the methodology used in the study. In contrast, eight studies mentioned that a random allocation of animals was made and that investigators were blinded (*n* = 8, 36.36%) (Figure 3).

Similar to our results, Toro et al. (2019), Sampaio et al. (2021), and Pina et al. (2021) demonstrated that preclinical studies in animals present insufficient information that implies judgment of domains. In contrast, domains that include allocation and blinding of investigators are reported in most studies included in systematic reviews (Toro et al., 2019; Pina et al., 2021; Sampaio et al., 2021).

Thus, there is a need for adequacy of new pre-clinical studies and reports using the criteria established in the Syrcle tool in order to improve the quality of studies and provide concrete evidence that can be reproduced in future clinical studies (Hooijmans et al., 2014).

### 3.6 Limitations

This review presented as a major limitation the impossibility of performing meta-analysis due to the high variability of the experimental protocols included in this study.

## 4 Conclusion

The results of this review suggest an antinociceptive potential of natural products from plants in CIPN, mainly because they are acting in different mechanisms of action (Dopamine D1 agonist, modulation of N and T calcium channels, inhibition of TRPV1), as well as direct action in oxidative stress and inflammatory mediators. We demonstrated that 21 out of 22 different natural products derived from plants found on the study presented a positive effect on reducing CIPN, these substances belong to different classes of secondary metabolites, such as alkaloids (Berberine, Evodiamine, Levo-tetrahydropalmatine, Matrine and Nicotine), cannabinoids (Cannabidiol and Delta-9-tetrahydrocannabinol), curcuminoids (Curcumin and Tetrahydrocurcumin), flavonoids (6-Methoxyflavanone, 6-Methoxyflavone, Icariin, Quercetin and Rutin), terpenes [Aucubin, Betulinic acid, (−)-Hardwickiic acid, Pedicularis-lactone, Viteoid I and Viteoid II] and xanthines (Propentofylline). Thus, being strategic compounds for the development of new therapeutic options for this multifactorial condition. However, more studies on safety and mechanisms of action are necessary.
