# Supplementary material for: Antinociceptive effect of plant-based natural products in chemotherapy-induced peripheral neuropathies: A systematic review
Source: Front Pharmacol. 2022 Sep 19;13:1001276. doi: 10.3389/fphar.2022.1001276 (PMC9527321; doi:10.3389/fphar.2022.1001276)
Supplement: Supplementary file 1 [file DataSheet1.DOCX]

Supplementary Material

**Antinociceptive effect of plant-based natural products in chemotherapy-induced peripheral neuropathies: A systematic review**

**Wagner Barbosa da Rocha Santos^a^, Lícia Tairiny Santos Pina^b^, Mairim Russo Serafini^a,b^, Adriana G. Guimarães^a,^***

^a^Graduate Program in Pharmaceutical Sciences, Department of Pharmacy, Federal University of Sergipe, São Cristóvão, Sergipe, Brazil.

^b^Graduate Program in Health Sciences, Federal University of Sergipe, São Cristóvão, Sergipe, Brazil.

*** Correspondence:**

Adriana Gibara Guimarães

adrianagibara@hotmail.com

**1. METHODS**

**1.1. Search strategy**

**Table S1:** Search strategy used in PubMed, Web of Science and Scopus databases.

| Data  Bases | Strategy | Results |
| --- | --- | --- |
| Pubmed | (((“chemotherapy-induced peripheral neuropathy”) OR (CIPN) OR (“peripheral neuropathy” AND cancer)) AND (((“Biological Products”) OR (“Biologic Products”) OR (“Products, Biological”) OR (“Natural Products”) OR (Biopharmaceuticals) OR (“Biological Drugs”) OR (“Drugs, Biological”) OR (Biologics) OR (Biologicals) OR (“Biologic Medicines”) OR (“Medicines, Biologic”) OR (“Biologic Pharmaceuticals”) OR (“Pharmaceuticals, Biologic”) OR (“Biologic Drugs”) OR (“Drugs, Biologic”) OR (“Biological Medicines”) OR (“Medicines, Biological”)) OR (((Terpenes) OR (Terpenoids) OR (Isoprenoids)) OR ((Flavonoids) OR (2-Phenyl-Chromenes) OR (“2 Phenyl Chromenes”) OR (2-Phenyl-Benzopyrans) OR (“2 Phenyl Benzopyrans”) OR (Bioflavonoids)) OR ((Coumarins) OR (Coumarines) OR (“1,2-Benzopyrone Derviatives”) OR (“1,2 Benzopyrone Derviatives”) OR (“Derviatives, 1,2-Benzopyrone”) OR (Benzopyran-2-ones) OR (“Benzopyran 2 ones”) OR (“Coumarin Derivatives”) OR (“Derivatives, Coumarin”) OR (1,2-Benzopyrones) OR (“1,2 Benzopyrones”) OR (1,2-Benzo-Pyrones) OR (“1,2 Benzo Pyrones”)) OR ((Xanthones) OR (“Xanthone Derivatives”) OR (“Derivatives, Xanthone”)) OR ((Chromones) OR (1,4-Benzopyrones) OR (“1,4 Benzopyrones”)) OR ((Lignans) OR (Neolignans)) OR ((Tannins) OR (“Tannic Acids”) OR (“Acids, Tannic”) OR (“Tannic Acid”) OR (“Acid, Tannic”)) OR ((Saponins) OR (Saponin)) OR ((Alkaloids) OR (“Plant Alkaloids”) OR (“Alkaloids, Plant”)) OR (Xanthines))) | 1473 |
| Web of Science | TS=((((Biological Products) OR (Biologic Products) OR (Products, Biological) OR (Natural Products) OR (Biopharmaceuticals) OR (Biological Drugs) OR (Drugs, Biological) OR (Biologics) OR (Biologicals) OR (Biologic Medicines) OR (Medicines, Biologic) OR (Biologic Pharmaceuticals) OR (Pharmaceuticals, Biologic) OR (Biologic Drugs) OR (Drugs, Biologic) OR (Biological Medicines) OR (Medicines, Biological)) OR (((Terpenes) OR (Terpenoids) OR (Isoprenoids)) OR ((Flavonoids) OR (2-Phenyl-Chromenes) OR (2 Phenyl Chromenes) OR (2-Phenyl-Benzopyrans) OR (2 Phenyl Benzopyrans) OR (Bioflavonoids)) OR ((Coumarins) OR (Coumarines) OR (1,2-Benzopyrone Derviatives) OR (1,2 Benzopyrone Derviatives) OR (Derviatives, 1,2-Benzopyrone) OR (Benzopyran-2-ones) OR (Benzopyran 2 ones) OR (Coumarin Derivatives) OR (Derivatives, Coumarin) OR (1,2-Benzopyrones) OR (1,2 Benzopyrones) OR (1,2-Benzo-Pyrones) OR (1,2 Benzo Pyrones)) OR ((Xanthones) OR (Xanthone Derivatives) OR (Derivatives, Xanthone)) OR ((Chromones) OR (1,4-Benzopyrones) OR (1,4 Benzopyrones)) OR ((Lignans) OR (Neolignans)) OR ((Tannins) OR (Tannic Acids) OR (Acids, Tannic) OR (Tannic Acid) OR (Acid, Tannic)) OR ((Saponins) OR (Saponin)) OR ((Alkaloids) OR (Plant Alkaloids) OR (Alkaloids, Plant)) OR (Xanthines))))  **AND**  TS=((("chemotherapy-induced peripheral neuropathy") OR (CIPN) OR (("peripheral neuropathy") AND (cancer)))) | 204 |
| Scopus | ( TITLE-ABS-KEY ( ( "chemotherapy-induced peripheral neuropathy" ) OR ( cipn ) OR ( "peripheral neuropathy" AND cancer ) ) AND ( TITLE-ABS-KEY ( ( ( "biological products" ) OR ( "biologic products" ) OR ( "products, biological" ) OR ( "natural products" ) OR ( biopharmaceuticals ) OR ( "biological drugs" ) OR ( "drugs, biological" ) OR ( biologics ) OR ( biologicals ) OR ( "biologic medicines" ) OR ( "medicines, biologic" ) OR ( "biologic pharmaceuticals" ) OR ( "pharmaceuticals, biologic" ) OR ( "biologic drugs" ) OR ( "drugs, biologic" ) OR ( "biological medicines" ) OR ( "medicines, biological" ) ) OR ( ( ( terpenes ) OR ( terpenoids ) OR ( isoprenoids ) ) OR ( ( flavonoids ) OR ( 2-phenyl-chromenes ) OR ( "2 phenyl chromenes" ) OR ( 2-phenyl-benzopyrans ) OR ( "2 phenyl benzopyrans" ) OR ( bioflavonoids ) ) OR ( ( coumarins ) OR ( coumarines ) OR ( "1,2-benzopyrone derviatives" ) OR ( "1,2 benzopyrone derviatives" ) OR ( "derviatives, 1,2-benzopyrone" ) OR ( benzopyran-2-ones ) OR ( "benzopyran 2 ones" ) OR ( "coumarin derivatives" ) OR ( "derivatives, coumarin" ) OR ( 1,2-benzopyrones ) OR ( "1,2 benzopyrones" ) OR ( 1,2-benzo-pyrones ) OR ( "1,2 benzo pyrones" ) ) OR ( ( xanthones ) OR ( "xanthone derivatives" ) OR ( "derivatives, xanthone" ) ) OR ( ( chromones ) OR ( 1,4-benzopyrones ) OR ( "1,4 benzopyrones" ) ) OR ( ( lignans ) OR ( neolignans ) ) OR ( ( tannins ) OR ( "tannic acids" ) OR ( "acids, tannic" ) OR ( "tannic acid" ) OR ( "acid, tannic" ) ) OR ( ( saponins ) OR ( saponin ) ) OR ( ( alkaloids ) OR ( "plant alkaloids" ) OR ( "alkaloids, plant" ) ) OR ( xanthines ) ) ) ) ) | 576 |
